# Isostatic Hot Pressed W–Cu Composites with Nanosized Grain Boundaries: Microstructure, Structure and Radiation Shielding Efficiency against Gamma Rays

**DOI:** 10.3390/nano12101642

**Published:** 2022-05-11

**Authors:** Daria I. Tishkevich, Tatiana I. Zubar, Alexander L. Zhaludkevich, Ihar U. Razanau, Tatiana N. Vershinina, Anastasia A. Bondaruk, Ekaterina K. Zheleznova, Mengge Dong, Mohamed Y. Hanfi, M. I. Sayyed, Maxim V. Silibin, Sergei V. Trukhanov, Alex V. Trukhanov

**Affiliations:** 1 Laboratory of Magnetic Films Physics, SSPA “Scientific and Practical Materials Research Centre of NAS of Belarus”, P. Brovki Str. 19, 220072 Minsk, Belarus; fix.tatyana@gmail.com (T.I.Z.); zheludkevich27@gmail.com (A.L.Z.); ir23.by@gmail.com (I.U.R.); bondruk625@gmail.com (A.A.B.); katenickerd@gmail.com (E.K.Z.); truhanov86@mail.ru (A.V.T.); 2 Laboratory of Single Crystal Growth, South Ural State University, Lenin Ave. 76, 454080 Chelyabinsk, Russia; 3 Frank Laboratory of Neutron Physics, Joint Institute for Nuclear Research, Joliot-Curie Str. 6, 141980 Dubna, Russia; vershinina@nf.jinr.ru; 4 Faculty of Natural and Engineering Sciences, Dubna State University, Universitetskaya Str. 19, 141980 Dubna, Russia; 5 Department of Micro- and Nanoelectronics, Belarusian State University of Informatics and Radioelectronics, P. Brovki Str. 6, 220013 Minsk, Belarus; 6 Department of Resource and Environment, Northeastern University, Wenhua Road 3-11, Shenyang 110819, China; mg_dong@163.com; 7 Institute of Physics and Technology, Ural Federal University, Mira Str. 19, 620002 Yekaterinburg, Russia; mokhamed.khanfi@urfu.ru; 8 Nuclear Materials Authority, El Maadi, Cairo P.O. Box 530, Egypt; 9 Department of Physics, Faculty of Science, Isra University, Al Hezam Road, Amman 1162, Jordan; dr.mabualssayed@gmail.com; 10 Department of Nuclear Medicine Research, Institute for Research and Medical Consultations, Imam Abdulrahman bin Faisal University, Dammam 31441, Saudi Arabia; 11 Scientific and Technological Park of Biomedicine, I.M. Sechenov First Moscow State Medical University, Bolshaya Pirogovskaya Str. 2/4, 119991 Moscow, Russia; sil_m@mail.ru; 12 Department of Electronic Materials Technology, National University of Science and Technology MISiS, Lenin Ave. 4/1, 119049 Moscow, Russia

**Keywords:** isostatic hot pressing, tungsten–copper composite, microstructure, structure, radiation shielding, gamma rays

## Abstract

The W–Cu composites with nanosized grain boundaries and high effective density were fabricated using a new fast isostatic hot pressing method. A significantly faster method was proposed for the formation of W–Cu composites in comparison to the traditional ones. The influence of both the high temperature and pressure conditions on the microstructure, structure, chemical composition, and density values were observed. It has been shown that W–Cu samples have a polycrystalline well-packed microstructure. The copper performs the function of a matrix that surrounds the tungsten grains. The W–Cu composites have mixed bcc-W (sp. gr. Im $\bar{3}$ m) and fcc-Cu (sp. gr. Fm $\bar{3}$ m) phases. The W crystallite sizes vary from 107 to 175 nm depending on the sintering conditions. The optimal sintering regimes of the W–Cu composites with the highest density value of 16.37 g/cm^3^ were determined. Tungsten–copper composites with thicknesses of 0.06–0.27 cm have been fabricated for the radiation protection efficiency investigation against gamma rays. It has been shown that W–Cu samples have a high shielding efficiency from gamma radiation in the 0.276–1.25 MeV range of energies, which makes them excellent candidates as materials for radiation protection.

## 1. Introduction

Currently, W–Cu composite materials are widely used in various fields of science and technology [1,2,3,4]. The number of materials that demonstrate excellent properties under extreme conditions, such as high voltage, extremely high temperatures, or hazardous radiation fields, typically include high-density and melting materials based on Nb, Mo, Ta, W, Re, and Bi [5,6,7,8,9]. Composites made of tungsten–copper (W–Cu) components have integrated properties that include low thermal expansion, high arc and corrosion resistance, high hardness and strength, and a high melting point [10,11,12,13]. The thermal and electrical conductivity of W–Cu alloys is strongly influenced by the microstructure and Cu content, as well as the mechanical strength of the W skeleton [14,15]. W–Cu composites demonstrate excellent electrical, mechanical, and thermal properties as described in references [3,6,10,11]. Because of their potential practical applications, tungsten–copper composites have received a lot of attention [1,6,16,17,18,19]. W–Cu composites are widely used in the electronic industry in the production of electrodes for welding and emission, high-voltage electrical contacts, microcircuit packages, and biomedical implants, and are promising for aerospace applications [20,21,22,23,24]. The composites based on W–Cu have a high shielding efficiency against electron and proton radiation from the Earth’s radiation belt [2]. It has been shown that the W–Cu composite has great attenuation properties against high-energy C^+^, Ne^+^, Ar^+^, Kr^+^, and Xe^+^ ions, which is very relevant for radiation-resistant chip package production [17]. Thus, obtaining composite materials based on W–Cu with the high-density values required for their use as shielding materials is a relevant task.

A homogeneous and dense structure is the paramount requirement to ensure excellent W–Cu alloy properties. Because of the hydrophobic behavior and the enormous difference in melting points between Cu (1085 °C) and W (3422 °C), the most conventional composite production technologies are powder metallurgy and high-temperature liquid sintering [25,26]. Nevertheless, it is an urgent task to synthesize the W–Cu composite of a high density with a complex shape and large area due to differences in the W and Cu properties using typical methods. 

Due to the mutual insolubility of W and Cu, powder metallurgy is the most widely used method for almost all composite fabrication, but this method does not guarantee the required density and mechanical properties of the W–Cu alloys [25,26,27]. To improve the relative density and wettability of copper and tungsten, a small amount of Co, Ni, Ti, Cr, or Fe is usually added to the W powder as sintering activators [28,29,30,31]. The isostatic cold pressing of W powder, combined with 1100 °C annealing for 2 h for the tungsten skeleton formation and subsequent copper infiltration at 1350 °C in a hydrogen atmosphere for 90 min, is used for the W–Cu composite fabrication [32]. The obtained composites have sufficient metallurgical bonding and a negligible number of pores, but there are two main disadvantages, such as multistage technology and the long duration of sintering. For the production of electrical contacts, W–Cu–Ni composites were manufactured using hot isostatic pressing combined with mechanical alloying [33]. Ni was added as an activator for the reduction of the sintering temperature of tungsten from 2800 to 1400 °C. The sintering process included two main stages: i. the consolidation of the milled powders (400 MPa at an automatic cold press); ii. the compactization at 1300 ± 10 °C and 1.5 h of maintaining time at 190 MPa. In other work [34], a combination of hot isostatic pressing and hot radial pressing was used for the mono-block plasma-facing components of the vertical target regions of the experimental advanced superconducting tokamak divertor. Another widely used method is to infiltrate liquid Cu into the porous W skeleton, but the functional properties are limited by powder agglomerates and porous defects [35]. Additive technologies, according to the ISO/ASTAM 52900 standard [36], are also applied for the composite fabrication. Three-dimensional printing technology is a layer-by-layer powder-based shaping and consolidation process. It provides an opportunity to obtain the W–Cu composites without geometric constraints or defects [25,37]. Another kind of additive manufacturing is a directed energy deposition consisting of the use of a high-energy laser, which irradiates and simultaneously heats a substrate and initial powder. The deposition process consists of creating a melt on a substrate and simultaneously feeding the powder into the melt [38,39,40]. In general, other additive techniques are used in the composite formation, such as powder bed fusion for the surface-modified copper powder production and subsequent laser-based additive manufacturing [41,42,43], binder jet printing for the tungsten-heavy alloying [44], and the extrusion of the material for the porous tungsten carbide composite fabrication [45], etc. 

To obtain a thin W–Cu alloy with low roughness, the magnetron co-sputtering method can be applied [46]. The copper electroplating of the initial tungsten powder followed by two hours of pressing at 850 °C and 100 MPa results in a high-density composite formation, but this process is limited by the difficulties of an electrochemical reaction and the long duration of sintering [47,48]. Chemical methods are used for the W–Cu alloy fabrication, but they are often difficult methods, are complex to control, and involve practically non-scalable technologies [49]. A spark plasma method using a combination of temperature, pressure, and an electric field is used to create a composite densification enhancer [50]. 

Regarding the composite material importance, the main purpose of the present study is to investigate the influence of sintering conditions on a new fast method based on isostatic hot pressing in order to obtain W–Cu composites with the necessary density values for shielding applications. In our recent paper [51], we reported the LAC and HVL for composite materials with a composition of W-75 wt.% Cu-25 wt.% and W-85 wt.% Cu-15 wt.%, while in the current paper, we extended our work on the W-85 wt.% Cu-15 wt.% composition and reported the features of the synthesis method, microstructure, structure, and shielding efficiency.

## 2. Materials and Methods

The W–Cu samples of two types were composites of W-85 wt.% and Cu-15 wt.% composition. Tungsten and copper powders were of analytical grade. The initial copper powder was obtained by purifying the material with 99.95 wt.% by electrolytic refining, followed by vacuum re-melting and high-temperature heating of the melt in order to obtain a substance containing substitutional and interstitial impurities at a level of <10^−5^ at.%. A planetary ball mill, the Fritsch Pulverisette (FRITSCH Laboratory Instruments, Idar-Oberstein, Germany), was used for the homogenization (mixing and milling) of the initial powders. The duration of grinding in a planetary ball mill was 4 h at a rotation speed of 300 rpm. The volume of the grinding jars was 250 mL. Grinding balls based on tungsten carbide were in the quantity of 15 balls. 

**Figure 1:**
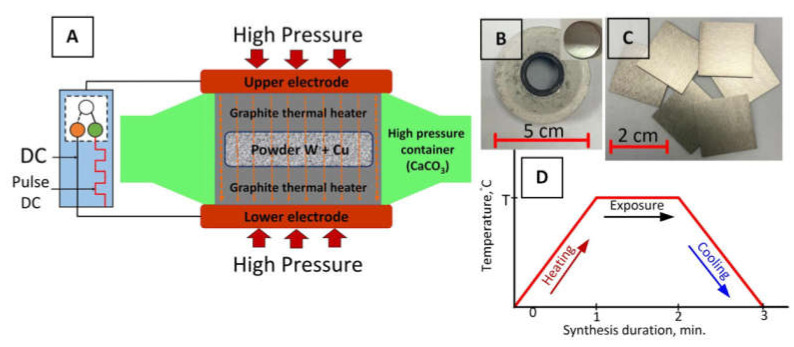
Schematic view of the isostatic hot pressing (**A**), sample preparation container with graphite heating element and W–Cu samples (insert) (**B**), radiation shields based on W–Cu composite with a 2.0 × 2.0 cm^2^ size and different thicknesses (**C**), and a schematic view of the sintering process (**D**).

An isostatic hot pressing method was used for the sample preparation (Figure 1A). A schematic view of the sintering process is shown in Figure 1D. The method of isostatic hot pressing consists of generating high pressure due to the spread of a high-pressure container (Figure 1B), which is carried out when the container with a sample is compressed in an apparatus due to inhibition of the spreading by the closing edges of the apparatus. High-temperature values are set by passing an electric current through the heating element system of the container, which is made of graphite-containing mixtures with low resistance, which makes it possible to obtain high temperatures in a small volume in a short period of time (Figure 1A). The mechanism of sintering consists of the simultaneous action of temperature and pressure on a closed volume. When exposed to high pressure, the intergranular distance decreases, and the particles of the mixture of powders (W and Cu) are compacted. As a result, the reaction area increases. In this case, the activation energy of the chemical reaction decreases. A mechanical collision of powder particles occurs, the process of mechanical activation takes place, and, as a result, the course of a chemical reaction accelerates. A distinctive feature of this method is that the heating and cooling of the sample occur under the constant influence of high pressure, which results in a high sintering rate. In our case, the sintering of samples is carried out within 3 min (Figure 1D), while in reference [52], a similar method is used, but with a duration of 180 min.

Initially, the W–Cu samples were fabricated in the form of tablets with a diameter of 2.6 cm and a thickness of 0.3 cm. Further, by mechanical processing, square samples with a 2.0 × 2.0 cm^2^ size were formed. Two types of samples were made: the first, six samples with a 0.27 ± 0.05 cm thickness for the investigation of the sintering process influence on the W–Cu structure, microstructure parameters, chemical composition, and density (effective and relative); the second, five samples prepared using optimized technology with 0.06, 0.09, 0.12, 0.15, and 0.27 ± 0.05 cm thicknesses for shielding properties measurements. All samples were polished and washed in ethanol before investigation. The relative error for temperature and pressure values during isostatic hot pressing is no more than 1% and 2.5%, respectively. Table 1 represents the information about the sintering regimes of the W–Cu composites.

Composite surface morphology and chemical composition studies were realized with the scanning electron microscope (SEM) Carl Zeiss EVO10 (Carl Ziess, Oberkochen, Germany) at an accelerating voltage of 20 kV in conjunction with the Oxford energy-dispersive X-ray (EDX) detector (Oxford Instruments NanoAnalysis, Wiesbaden, Germany). A backscattered electron detector (BSD) was used for a more detailed study of the surface morphology and contrast chemical composition. The effective density of a sintered composite was measured using Archimedes’ principle [13]. A statistical grain analysis was performed using the standard method described in detail in reference [7]. Statistical analysis of grain size using the software SmartSEM (Carl-Zeiss, Oberkochen, Germany) was performed for at least three SEM images.

The X-ray diffraction method (XRD) was applied to the evaluation of the crystal structure of synthesized W–Cu composites. The PANalytical EMPYREAN (Malvern Panalytical Ltd., Malvern, United Kingdom) powder diffractometer with CuKα radiation was used for XRD measurements. The diffraction lines were recorded for 2Θ = 20–106° with a step of 0.02°. The observed diffraction peaks were corrected by splitting into the peaks diffracted by Cu-Kα1 and Cu-Kα2 radiations. The peak profiles were fitted with the pseudo-Voigt function. The instrumental line broadening was evaluated by using the standard reference material of LaB6. The Williamson–Hall approach was used to estimate the crystallite size of tungsten.

**Figure 2:**
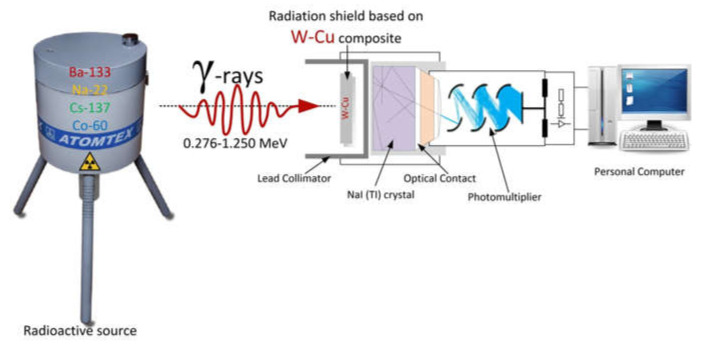
The scheme of radiation protection efficiency evaluation.

In order to assess the radiation shielding performance of the prepared composites, the radiation protection efficiency (RPE) was evaluated. For this aim, the following radioactive sources were used: Ba-133, Na-22, Cs-137, and Co-60. The intensities of the emitted photons (*I*_0_) and the photons that penetrated through composites (*I*) were estimated using the gamma-ray transmission method [53]. The transmitted photons were detected using a scintillation Bicron detector coupled with a 76 × 76 mm^2^ NaI (Tl) crystal. An aluminum block was used to encase the detector crystal and photomultiplier tube. In order to protect against the induced X-rays, the detector and amplifier were insulated with a Cu plate with a 0.6 mm thickness. Additionally, the detector was protected from ambient radiation by a 5 cm-thick layer of lead. The Accuspec card was linked to the detector. The measurements were conducted at room temperature. The Accuspec NaI detector with 2k onboard ADC, Amp, and HVPS with 2k channel memory linked to the PC board was used for the experiments (Figure 2).

The radiation protection efficiency (RPE) can be determined via the following equation:

(1) $$\mathrm{RPE}=(1-\frac{I}{I_{0}})\;\times \;100\%$$

## 3. Results and Discussion

**Figure 3:**
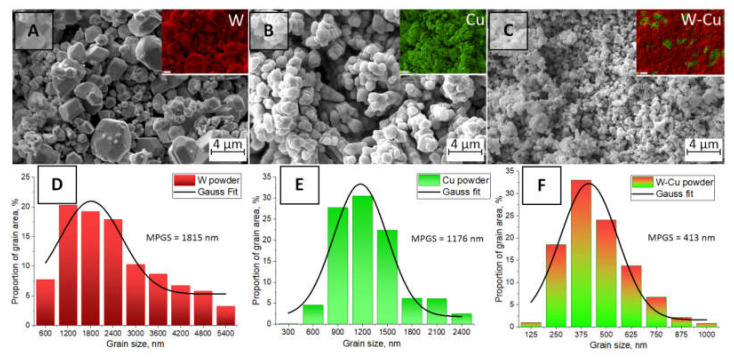
SEM images of the initial W powder (**A**), Cu powder (**B**), mixed and milled W–Cu powder (**C**), and corresponding grain size distribution (**D**–**F**). Inserts: EDX maps of elemental distribution.

The morphology of the initial W, Cu, and mixed and milled W–Cu powders was investigated (Figure 3). Tungsten powder has oval-shaped grains with well-defined boundaries [54]. It can be seen that copper powder has a dendritic morphology, which is typical for electrolytic powders. After mixing and milling, the powder has a fine-grained microstructure. However, as can be seen from the EDX results (Figure 3C), powder pretreatment does not promote the uniform mixing of the components of the W and Cu powder, since there are still areas with agglomerated Cu grains. Obviously, in order to avoid this unevenness in the future, other grades of copper powder that have a more finely dispersed structure should be used. 

The analysis of the grain size distribution shown in Figure 3D–F demonstrates the fraction of the individual grains of the powders and their size changes. The data collection was performed on at least three SEM images for the best statistical calculation. Tungsten grains smaller than 1.2 µm occupy about 20% of the powder surface area. The value of the most probable grain size (MPGS) according to the Gauss fitting corresponds to 1.82 µm. In the case of copper initial powder, the grains with sizes of less than 1.2 µm occupy about 30% of the whole investigated area, and MPGS becomes 1.18 µm. An interesting fact is that after the simultaneous mixing and milling of the two-component powder, the grains become fine-grained and more than 32% of the grains have a size of less than 375 nm when the MPGS is 413 nm. 

**Figure 4:**
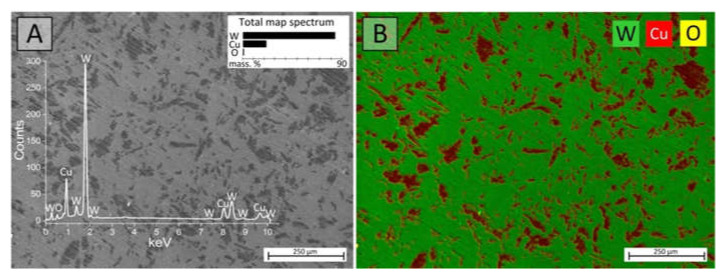
SEM image (**A**) and EDX map (**B**) of the W–Cu composite sample.

All W–Cu samples before SEM investigations were polished using a FORCIPOL 202 (Metkon Instruments Inc., Bursa, Turkey) grinding and polishing machine for the planarization and smoothness of the composite surface since the samples initially had a high roughness. An EDX study showed that composite samples have a W-85 wt.% and a Cu-15 wt.% composition. The chemical composition map of the unpolished Sample 3 of the I type showed the presence of mostly two main elements (Figure 4): tungsten (green color) and copper (red color). An insignificant amount of oxygen (yellow color) is also noted (less than 1 wt.%). It may be associated with the post-sintering oxidation of the composite.

**Figure 5:**
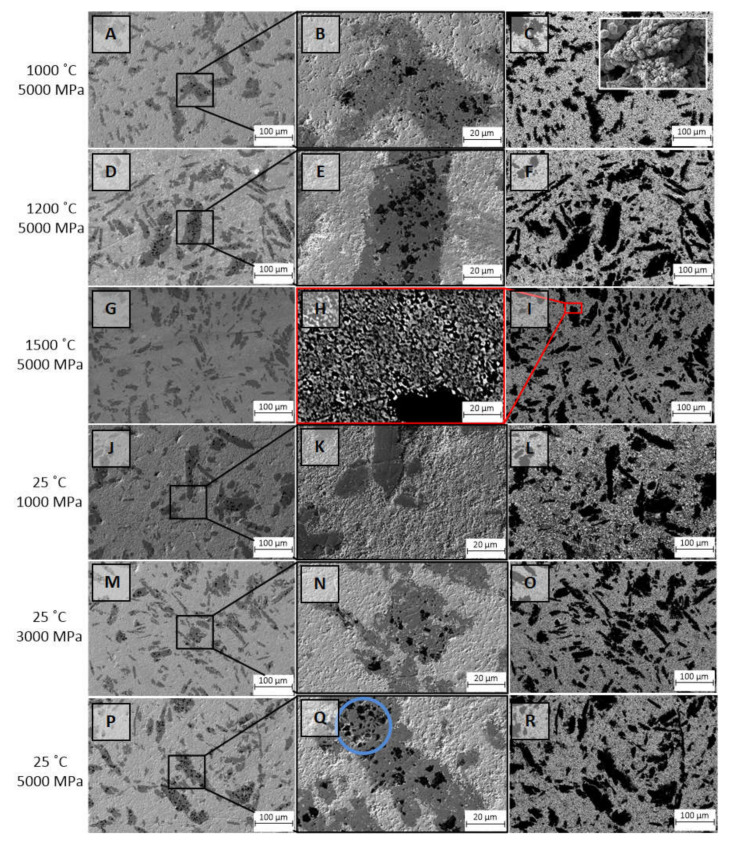
SEM images of the W–Cu composite samples of I type obtained in different sintering conditions and corresponding enlarged images: **A**–**C**—Sample 1, **D**–**F**—Sample 2, **G**–**I**—Sample 3, **J**–**L**—Sample 4, **M**–**O**—Sample 5, and **P**–**R**—Sample 6. Insert: SEM image of the initial copper powder used for the sample preparation.

The results of the microstructure investigations of the fabricated W–Cu samples are presented in Figure 5. All samples have a polycrystalline microstructure. It is seen that a combination of sintering conditions, such as high pressure and temperature, leads to the formation of dense composites with a well-packed microstructure, which is typical for Samples 1, 2, and 3. The number of pores decreases as the sintering temperature increases from 1000 to 1500 °C, whereas using only high pressures at room temperature results in the formation of composites with a loose powdery microstructure (Figure 5K,N,Q). SEM investigations using the BSD detector (Figure 5C,F,I,L,O,R) showed that the composite consists of two phases—copper (black) and tungsten (white). These contrast images show that the composite has nanosized grain boundaries, which are clearly visible in Figure 5H. The copper phase consists of two phases: a coarse-grained one with an oblong shape and a fine-grained one surrounding the tungsten grains. Moreover, it can be seen from Figure 5H that copper with grain sizes of hundreds of nm performs the function of a matrix that surrounds tungsten grains. However, all SEM images presented in Figure 5 showed the presence of large Cu phases that obviously agglomerated from fine-grained copper grains, and preliminary treatment did not contribute to their milling and mixing. It can be seen that Cu grains have black regions that are inclusions of the abrasive material (silicon carbide) that was used to grind the composites before the SEM investigation (blue circle in Figure 5Q).

**Figure 6:**
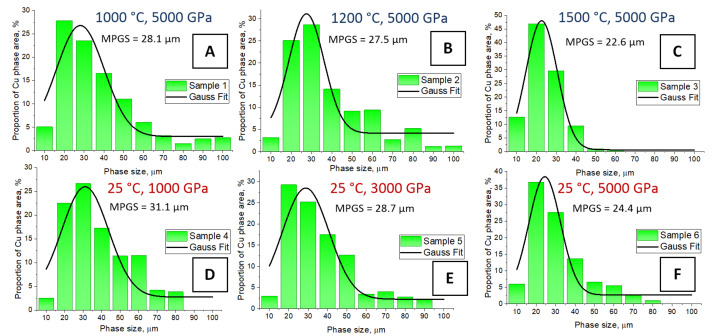
Distribution of the Cu phase sizes of the W–Cu samples of the I type: (**A**)—Sample 1, (**B**)—Sample 2, (**C**)—Sample 3, (**D**)—Sample 4, (**E**)—Sample 5, and (**F**)—Sample 6.

It is noteworthy that the samples after polishing have abrasive inclusions in the agglomerated Cu grains, while they are not observed in the samples before polishing (Figure 4) and in the polished samples synthesized at 1500 °C and 5000 MPa. Analyzing the SEM images, we concluded that the sizes of the copper phases change for each type of sample during sintering. The calculation results for the size distribution are shown in Figure 6. It is seen that for Samples 1–3 with a fixed pressure of 5000 MPa, a decrease in the size of the copper phase from 28.1 to 22.6 µm is observed with an increase in the sintering temperature up to 1500 °C. The same tendency is noticed for samples 4–6, obtained at room temperature with varying pressure values. However, an increase in the Cu phase size is observed as the pressure decreases from 5000 to 1000 MPa. We believe that the sintering of composites with the combined effect of high temperatures and pressures leads to better compaction of the initial powders and the fusion of the Cu phase with tungsten grains as a result of isostatic hot pressing, which correlates well with the SEM images. 

**Figure 7:**
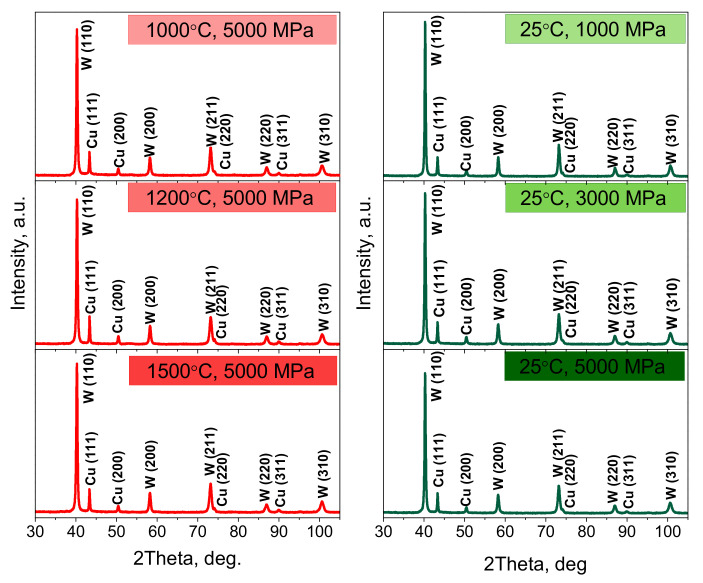
XRD patterns of W–Cu composite samples obtained in different sintering conditions.

According to the XRD investigation (Figure 7), the phase composition of all sintered W–Cu composites is represented by a mixture of bcc-W (sp. gr. Im $\bar{3}$ m, ICSD ref. code 98-004-4393) and fcc-Cu (sp. gr. Fm $\bar{3}$ m, ICSD ref. code 98-062-7113). No other phases are detected. The crystal lattice parameters of copper and tungsten in all studied states were 3.616 ± 0.001 Å and 3.167 ± 0.001 Å, respectively.

The estimates carried out by the Williamson–Hall approach make it possible to assess the tendency of changes in the crystallite sizes depending on the sintering conditions. Table 2 shows that the size of the crystallites in tungsten decreases with an increase in the sintering temperature from 1000 to 1500 °C under conditions of a pressure of 5000 MPa. Crystallite sizes remain practically unchanged during sintering at room temperature. Strains in all investigated states are approximately at the same level ~3 × 10^−4^.

It is known that crystallite sizes determined by XRD are generally smaller than grain sizes estimated from SEM images. This is due to the fact that the XRD method determines the size of the coherently diffracting domains (crystallites) that make up individual grains observed by the SEM method.

**Figure 8:**
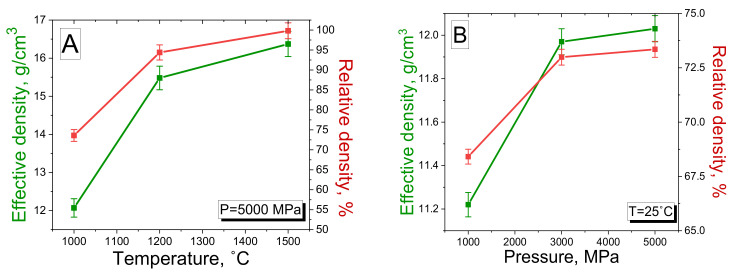
The dependence of effective and relative densities of the W–Cu composite on temperature (**A**) and pressure (**B**) values.

The effective and relative density evaluations showed the different behavior of these parameters during the sintering process (Table 3, Figure 8). 

The density values increase from 12.07 to 16.37 g/cm^3^ (for effective) and from 73.59 to 99.82% (for relative) as the sintering temperature and pressure increase from 1000 to 1500 °C and 5000 MPa, respectively. The opposite situation is observed for Samples 4–6, synthesized at a fixed temperature of 25 °C and with an increase in pressure from 1000 to 5000 MPa (Figure 8B). The density values rise slightly as pressure rises, from 11.22 to 12.03 g/cm^3^ (68.41–73.35% for relative density). Thus, we can conclude that the optimal conditions for the sintering of the W–Cu composites with the highest density values are a combination of both high pressure and temperature. Therefore, the second type of samples for studying the RPE was obtained at 1500 °C and 5000 MPa since such sintering conditions proved the composite fabrication with the maximum density value.

**Figure 9:**
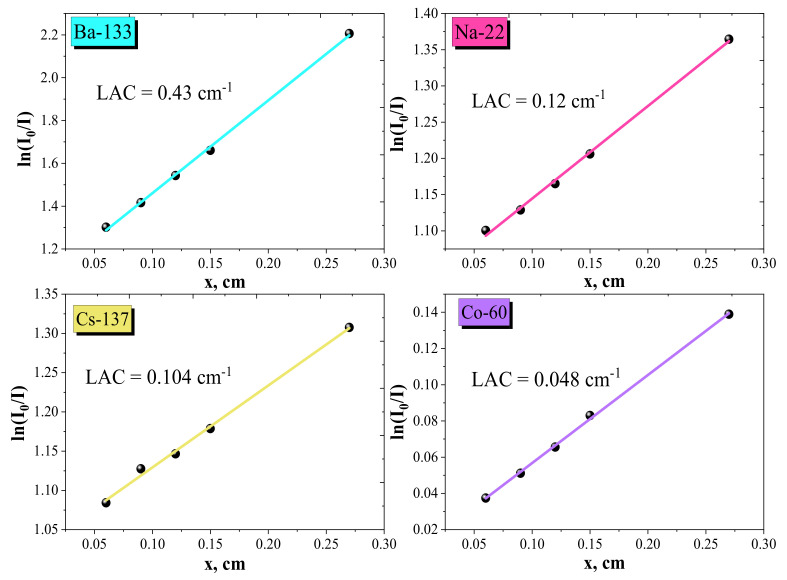
The variation of ln(*I*_0_/*I*) with the thickness (*x*, cm) of the W–Cu composites at different incident gamma photon energies.

Figure 9 displays the variation of ln(*I*_o_/*I*) with the thickness (*x*, cm) of the investigated W–Cu composites at various incident photon energies. The slope of Figure 9 is called the linear attenuation coefficient (LAC), which is estimated, as follows (in cm^−1^): LAC = ln ((*I*_o_/*I*)/*x*). From Figure 9, the deviation between the LAC values at various photon energies was detected. The highest value of LAC (0.43 cm^−1^) is balanced at low photon energy (Ba-133, 0.266 MeV) and the lowest (0.048 cm^−1^) is found at photon energy (Co-60, 1.25 MeV). This indicates the variation of LAC is due to the interaction of gamma radiation with the investigated W-85 wt.% and Cu-15 wt.% composites. At low energy, the predominant interaction is the photoelectric effect (PE), while at increasing gamma energy, the interaction will be the Compton scattering (CS). Therefore, the cross-section of the CS varies directly with the incident gamma energy (*σ_com_ α E*^−1^).

**Figure 10:**
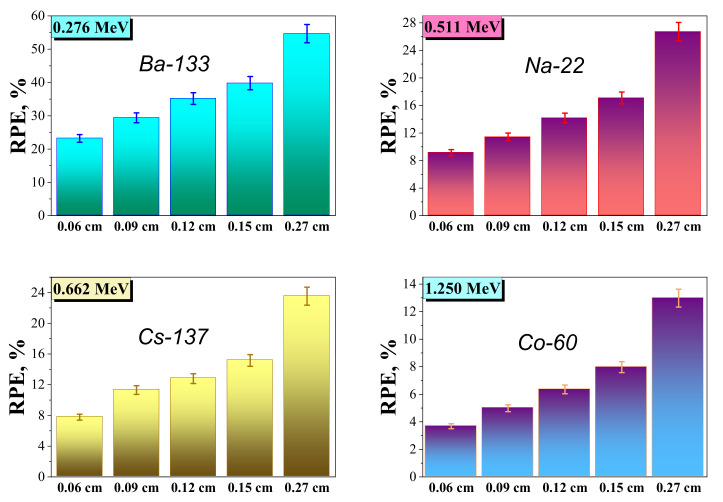
RPE values of the W–Cu composite with different thicknesses depending on the incident gamma photon energy.

Figure 10 represents the results of the RPE for the W–Cu composites at the examined energies and for the 0.06–0.27 cm thicknesses. It is seen that the growth of the thickness from 0.06 cm to 0.27 cm leads to an increase in the RPE values for the examined W–Cu composite at an identified photon energy. 

These results indicate that a thin W–Cu composite sample is required to shield the low-energy photons, while this thin sample cannot provide enough protection from the high-energy photons. Accordingly, we need a thick W–Cu composite sample to provide greater protection from the radiation that possesses high energy. For the tungsten–copper composite with a thickness of 0.06 cm, the RPE is 23.2%. This value is enhanced to 29.4% for a thickness of 0.12 cm, also increases to 35.2% for a thickness of 0.15 cm, and becomes 54.7% for a thickness of 0.27 cm. This means that if a layer of composite with a thickness of 0.06 cm is exposed to photons with an energy of 0.276 MeV, then this layer can only attenuate about 23% of the incoming photons, and most of the photons (about 77% of the incoming photons) can penetrate this sample. While the W–Cu sample with a thickness of 0.27 cm is exposed to the photons (with an energy of 0.266 MeV), half of the incoming photons will be absorbed by this layer and only 45.3% can penetrate this layer. Therefore, the thickness strongly affects the shielding performance of the W–Cu composite. As demonstrated in Table 4, at a specific thickness, we can see that the RPE for the W–Cu composite also depends on the energy. 

As the energy increases, the RPE decreases, which means that the high-energy photons can easily penetrate the composite. Thus, for a layer with a thickness of 0.27 cm, the RPE decreases from 54.7% at 0.266 MeV to 23.5% at 0.662 MeV and to 13% at 1.25 MeV. This suggests that the composite of W-85 wt.% and Cu-15 wt.% composition has better attenuation performance when exposed to low-energy radiation. According to the previous findings, a composite with a high thickness can be developed for radiation shielding applications.

## 4. Conclusions

A new fast isostatic hot pressing method for the sintering of W–Cu composites with nanosized grain boundaries was proposed. The use of this fast method contributed to a significant reduction in sintering duration of up to 3 min in comparison with the information available in the literature about other techniques. The influence of the sintering process on the microstructure and the effective and relative density has been revealed. An optimization of the sintering process allowed for the fabricating of W–Cu composites with a 0.06–0.27 cm thickness and a 99.82 relative density for the RPE against gamma-ray measurements. 

A change in the composite microstructure from loose and powdery to well-packed with a combined increase in temperature and pressure values was revealed. Energy-dispersive X-ray analysis evaluated the presence of the main two components, such as tungsten and copper, with an insignificant amount of oxygen. W–Cu composites have a polycrystalline structure with mixed bcc-tungsten (sp. gr. Im $\bar{3}$ m) and fcc-copper (sp. gr. Fm $\bar{3}$ m) phases. It has been shown that all studied samples have W crystallite sizes ranging from 107 to 175 nm. It has been observed that the effective density rises from 12.07 to 16.37 g/cm^3^ with temperature increases up to 1500 °C at a pressure of 5000 MPa. The RPE of the W–Cu composite samples against gamma rays was measured using Ba-133, Na-22, Cs-137, and Co-60 sources. The highest value of LAC (0.43 cm^−1^) was found at low photon energy (Ba-133, 0.266 MeV), while the lowest (0.048 cm^−1^) was reported at a photon energy of 1.25 MeV. It has been shown that the rise in the RPE occurs as the sample thickness increases. The W–Cu composite sample with a 0.27 cm thickness is more effective for all the investigated energies. From a practical point of view, samples with a thickness of more than 0.2 cm are recommended for use as radiation protection materials against gamma rays.

## Figures and Tables

**Table 1:** Sintering regimes of the W–Cu composite samples.

Sample Type	Sample No.	Composition, wt.%	Temperature (T), °C	Pressure (P), MPa	Thickness, cm
I	1	W_85_Cu_15_	1000	5000	0.27
I	2	W_85_Cu_15_	1200	5000	0.27
I	3	W_85_Cu_15_	1500	5000	0.27
I	4	W_85_Cu_15_	25	1000	0.27
I	5	W_85_Cu_15_	25	3000	0.27
I	6	W_85_Cu_15_	25	5000	0.27
II	1	W_85_Cu_15_	1500	5000	0.06
II	2	W_85_Cu_15_	1500	5000	0.09
II	3	W_85_Cu_15_	1500	5000	0.12
II	4	W_85_Cu_15_	1500	5000	0.15
II	5	W_85_Cu_15_	1500	5000	0.27

**Table 2:** The values of W crystallite sizes obtained by the Williamson–Hall approach.

Sample No.	Crystallite Size, nm
1	175
2	152
3	103
4	128
5	127
6	107

**Table 3:** The effective and relative densities of the I type of W–Cu composite samples.

Sample No.	Effective Density, g/cm^3^	Relative Density, %
1	12.07	73.59
2	15.48	94.39
3	16.37	99.82
4	11.22	68.41
5	11.97	72.99
6	12.03	73.35

**Table 4:** Radiation protection efficiency of the W–Cu composites with various thicknesses.

Energy, MeV	RPE, %				
	0.06 cm	0.09 cm	0.12 cm	0.15 cm	0.27 cm
**0.276**	23.2 ± 1.20	29.4 ± 1.40	35.2 ± 1.75	39.8 ± 1.95	54.7 ± 2.70
**0.662**	7.8 ± 0.40	11.3 ± 0.50	12.8 ± 0.64	15.2 ± 0.75	23.5 ± 1.15
**0.511**	9.1 ± 0.45	11.4 ± 0.55	14.2 ± 0.70	17.1 ± 0.85	26.7 ± 1.30
**1.25**	3.7 ± 0.18	5 ± 0.25	6.3 ± 0.30	8 ± 0.40	13 ± 0.65

## Data Availability

The data presented in this study are available on request from the corresponding authors.
